# Sulfotyrosine residues: Interaction specificity determinants for extracellular protein–protein interactions

**DOI:** 10.1016/j.jbc.2022.102232

**Published:** 2022-07-05

**Authors:** Valley Stewart, Pamela C. Ronald

**Affiliations:** 1 Department of Microbiology & Molecular Genetics, University of California, Davis, USA; 2 Department of Plant Pathology, University of California, Davis, USA; 3 Genome Center, University of California, Davis, USA

**Keywords:** C–C chemokine receptor type 5, cytokine, follicle-stimulating hormone, leucine-rich repeat, peptide hormone, plant hormone, post-translational modification, protein–protein interaction, thrombin, tyrosine sulfation, CCK, cholecystokinin, CCL5, CC-type chemokine ligand 5, CCR5, C–C chemokine receptor type 5, CD4i, CD4-induced, chemokine, chemotactic cytokine, CHIPS, Chemotaxis inhibitory protein of *Staphylococcus aureus*, CXCR4, CXC-type chemokine receptor 4, DTI, direct thrombin inhibitor, FVIII, factor VIII, GAG, glycosaminoglycan, gp, glycoprotein, gp120, glycoprotein spike 120 kDa subunit, GPCR, G protein-coupled receptor, GPIHBP1, glycosylphosphatidylinositol-anchored high-density lipoprotein binding protein 1, HCD3, heavy chain with unusually long and diverse sequences for the third complementarity-determining region, LRR, leucine-rich repeat, MS, mass spectrometry, PAP, 3′-phosphoadenosine 5′-phosphate, PAPS, 3′-phosphoadenosine 5′-phosphosulfate, PSGL-1, P-selectin glycoprotein ligand-1, PSK, phytosulfokine, PSY, plant peptide containing sulfated tyrosine, PTM, post-translational modification, pTyr, phosphotyrosyl, RaxX, required for activation of Xa21, RiPP, ribosomally synthesized and post-translationally modified peptide, RGF, root meristem growth factor, SH2, src homology 2, sTyr, sulfotyrosyl residue, TPST, tyrosyl-protein sulfotransferase, VISTA, V-domain immunoglobulin suppressor of T-cell activation

## Abstract

Tyrosine sulfation, a post-translational modification, can determine and often enhance protein–protein interaction specificity. Sulfotyrosyl residues (sTyrs) are formed by the enzyme tyrosyl-protein sulfotransferase during protein maturation in the Golgi apparatus and most often occur singly or as a cluster within a six-residue span. With both negative charge and aromatic character, sTyr facilitates numerous atomic contacts as visualized in binding interface structural models, thus there is no discernible binding site consensus. Found exclusively in secreted proteins, in this review, we discuss the four broad sequence contexts in which sTyr has been observed: first, a solitary sTyr has been shown to be critical for diverse high-affinity interactions, such as between peptide hormones and their receptors, in both plants and animals. Second, sTyr clusters within structurally flexible anionic segments are essential for a variety of cellular processes, including coreceptor binding to the HIV-1 envelope spike protein during virus entry, chemokine interactions with receptors, and leukocyte rolling cell adhesion. Third, a subcategory of sTyr clusters is found in conserved acidic sequences termed hirudin-like motifs that enable proteins to interact with thrombin; consequently, many proven and potential therapeutic proteins derived from blood-consuming invertebrates depend on sTyrs for their activity. Finally, several proteins that interact with collagen or similar proteins contain one or more sTyrs within an acidic residue array. Refined methods to direct sTyr incorporation in peptides synthesized both *in vitro* and *in vivo*, together with continued advances in mass spectrometry and affinity detection, promise to accelerate discoveries of sTyr occurrence and function.

Post-translational modifications (PTMs) influence protein activity in many ways. Thus, learning how these modifications act singly and in combination is important for understanding protein function (1, 2). One PTM increasingly recognized as critical for diverse extracellular interactions in animals, plants, and certain bacteria is tyrosine O-sulfation, forming the sulfotyrosyl residue (sTyr; Tys in the three-letter code used for structural models). Sulfation is catalyzed during Golgi transit by tyrosyl-protein sulfotransferase (TPST) and therefore occurs only in secreted and membrane-spanning proteins (3).

sTyr was discovered nearly 60 years ago (4), but detecting and documenting this modification remains challenging such that the extent of sTyr occurrence has been incompletely defined (5, 6, 7). Although the sTyr sulfate linkage is generally stable in weak acid, it is cleaved by the strong acid used in the Edman degradation method used to determine protein sequences (6, 8). Compounding the difficulty, routine mass spectrometry (MS) methods do not reliably detect sTyrs (9), so specialized protocols are being developed to minimize sulfate loss during peptide ionization and fragmentation (7, 10, 11, 12, 13, 14, 15, 16).

Research on phosphotyrosyl (pTyr) occurrence and function relies in part on a variety of anti-pTyr antibodies, some with broad recognition and others specific for pTyr residues in defined sequence contexts (17). A commercially available anti-sTyr monoclonal antibody binds sTyrs with high affinity regardless of flanking sequence and discriminates between peptide sTyr and pTyr residues by several 100-fold (18). Nevertheless, this reagent has been used sparingly in sTyr research (11). More recently, two antibodies were identified that may discriminate between different chemotactic cytokine (chemokine) CC-type chemokine receptor 5 (CCR5) sulfoforms (19). It therefore may be feasible to isolate sequence-specific anti-sTyr antibodies to monitor individual sulfation sites.

Systems-level characterization of pTyr residues often employs initial affinity enrichment with a broad-specificity anti-pTyr antibody (20). High-affinity pTyr-binding src homology 2 (SH2) domains (approximately 100 residues) can provide an inexpensive alternative (20). Therefore, SH2 domains with high affinity and specificity for sTyr may offer a useful means for identifying and enriching sTyr-containing proteins (21, 22).

Finally, early research on sTyr occurrence and function was constrained by the availability of synthetic sulfopeptides for use in binding assays. No more than two sTyrs could be incorporated in a peptide (23), and some studies substituted pTyr for sTyr (24). Today, homogeneous sulfopeptides can be synthesized *in vitro* with up to three sTyrs (25, 26, 27), and sulfonyl analogs of sTyr provide increased stability (28). Separately, sulfopeptides with up to five sTyrs in all combinations have been synthesized in *Escherichia coli* strains that have an expanded genetic code, in which an amber (UAG)-reading tRNA is charged with exogenous sTyr (29, 30, 31, 32). A similar system decodes amber as sTyr in mammalian cells (33), providing the possibility for functionally analyzing individual sTyrs *in vivo*.

## Why sTyr?

Consider sTyr in contrast to the familiar pTyr residue. Both sulfation and phosphorylation add a functional group that is fully ionized at neutral pH and consequently results in increased side-chain polarity (34). However, sTyr makes weaker hydrogen bonds because of its lesser charge (–1 *versus* –2) and smaller dipole moment (34).

Functions for pTyr have been studied extensively: its critical role in cellular growth control was discovered in large part from analyzing tumor virus–encoded oncogenic proteins (35). Tyrosyl protein kinase activity serves as “writer” for signaling, whereas separate phosphoprotein phosphatase activity serves as “eraser.” For signal propagation, small pTyr-binding domains such as SH2 provide “reader” function for multidomain output complexes (36, 37). By contrast, sTyr is a long-lived PTM (38), and in most cases, the sulfate ester likely is stable in physiological conditions (6). Neither “reader” (*e.g.*, portable sTyr-binding domains) nor “eraser” (*e.g.*, sulfoprotein sulfatase) components are known.

The sTyr potentiates protein–protein interactions through dual means: the sulfate group, which can make multiple electrostatic interactions to basic Arg or Lys residues in the binding partner; and the Tyr aromatic ring, which engages in both nonpolar and stacking interactions with diverse binding partner residues. Although pTyr can at least partially replace sTyr for certain peptide–peptide interactions (24, 39, 40), the sTyr sulfate makes distinct ionic contacts (41, 42) and therefore in most cases provides a unique *in vivo* interaction specificity determinant.

**Figure 1:**
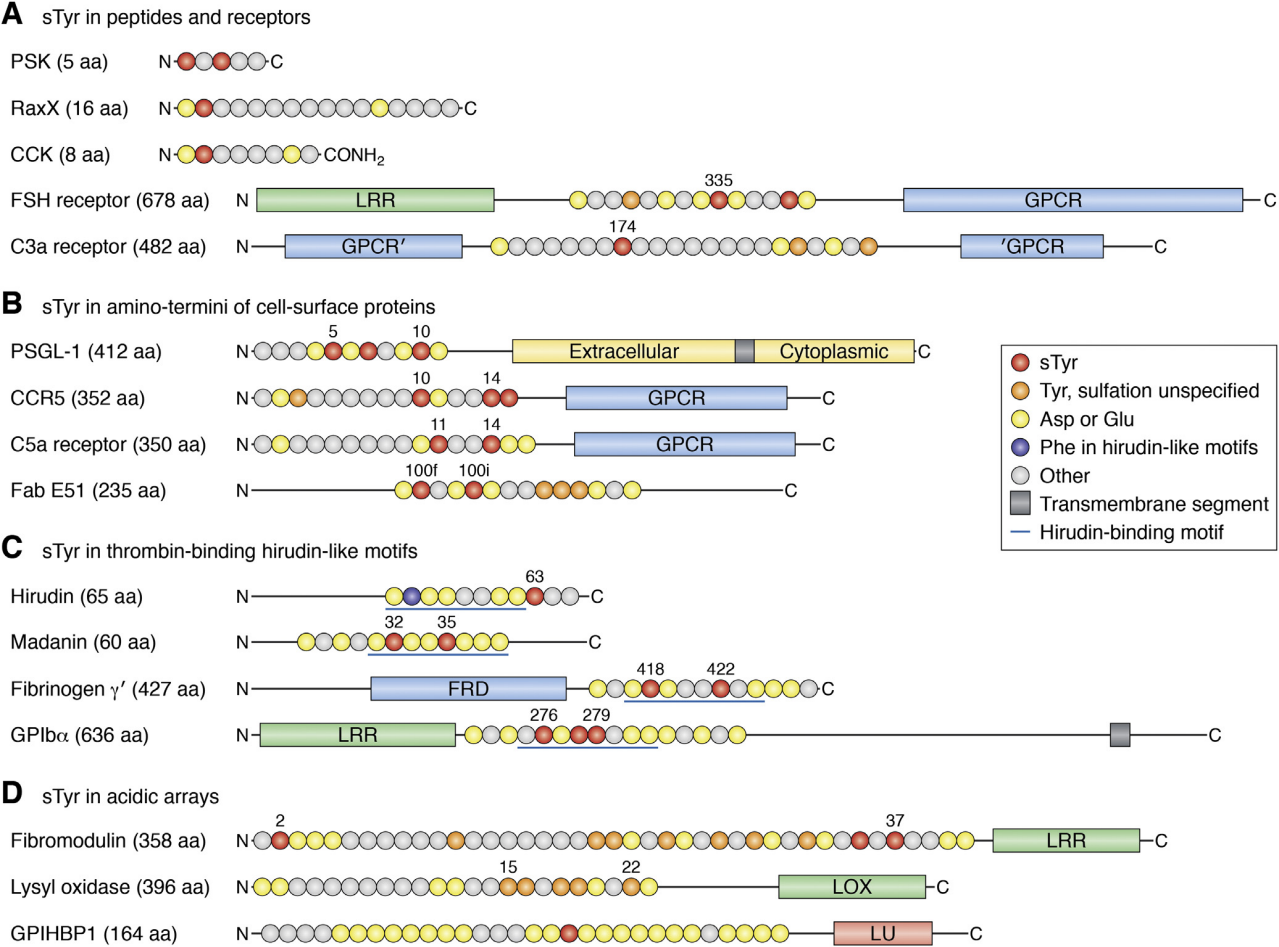
**Amino acid sequence contexts for representative sTyrs.** Primary structures of sTyr-containing proteins are shown schematically with major domains drawn approximately to scale. Segments with sTyrs are depicted as *circles*, each corresponding to a different amino acid, and colored according to the key. Proteins and peptides are described in the text. The amino and carboxyl termini are denoted N and C, respectively, and numbers indicate residue position in the sequence. *A*, sTyr in peptides and receptors. The CCK-8 Phe-amide terminus is denoted CONH_2_. The sTyr-containing sequence in the C3a receptor is in a loop within the seven transmembrane GPCR domain. *B*, sTyr in amino termini of cell-surface proteins. *C*, sTyr in hirudin-like motifs. The *blue lines* denote the eight-residue hirudin-like motif. *D*, sTyr in acidic arrays. CCK-8, cholecystokinin 8; GPCR, G protein–coupled receptor; sTyr, sulfotyrosyl residue.

sTyrs are documented in a variety of extracellular proteins (3, 9, 27, 43, 44, 45, 46) and currently are known to occur predominately in four broad contexts (Fig. 1): (a) in peptide hormones or their receptors, almost all with a single sTyr; (b) in conformationally flexible segments at the aminotermini of cell-surface proteins, most with two or three sTyr clustered within a span of six residues; (c) in conserved sequences (hirudin-like motifs) that interact with thrombin, all with two or three sTyrs clustered within a span of six residues; and (d) in tracts of acidic residues at the aminotermini of certain secreted proteins. Thus, sTyr-based interactions usually involve relatively short protein segments, often within large multidomain proteins.

Recent discoveries of previously unknown sTyr occurrence (47, 48) and functions (26, 49, 50, 51) show that the repertoire of known sulfoproteins will continue to expand. Here, we first describe the enzyme responsible for sTyr formation and then review examples that illustrate each of these four sTyr contexts (Fig. 1). We finish the article by considering some challenges and opportunities for future sTyr research.

## TPST

**Figure 2:**
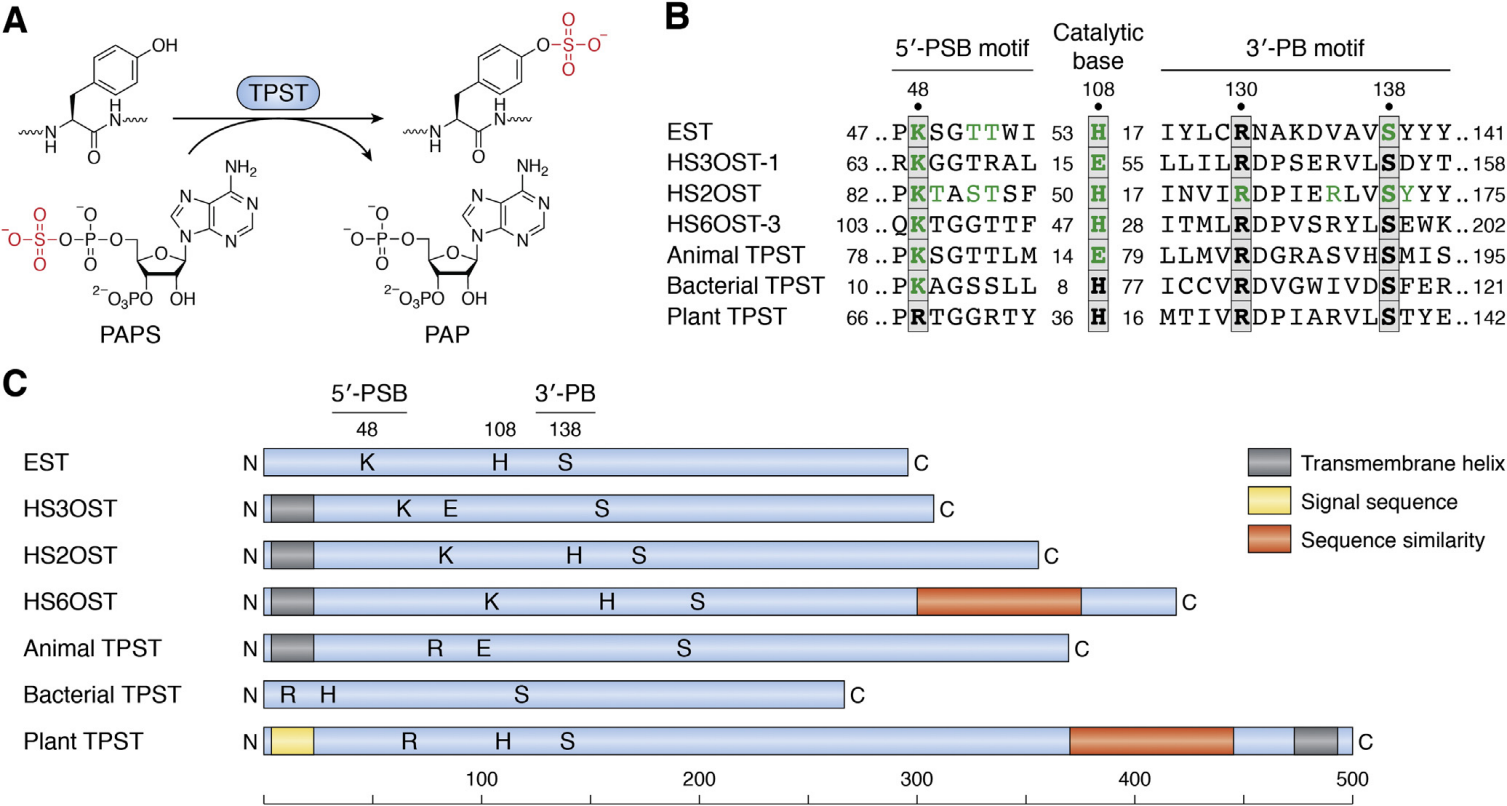
**Tyrosyl-protein sulfotransferase (TPST).***A*, tyrosyl-protein sulfotransferase catalyzes the transfer of sulfate from the universal sulfate donor PAPS (3′-phosphoadenosine 5′-phosphosulfate) to the hydroxyl group of a peptidyl-tyrosine residue to form a tyrosyl O4-sulfate ester and PAP (3′-phosphoadenosine 5′-phosphate). Redrawn from Ref. (5). *B*, sequence motifs from representative sulfotransferases. Substitutions at residues highlighted in *green* strongly decrease enzyme activity. Numbers at the *left* and *right* ends show positions within the sequence; internal numbers show the number of residues between elements. *C*, primary structures of representative sulfotransferases, drawn approximately to scale. *Letters* show the approximate positions of the 5′-PSB (K or R), catalytic base (H or E), and 3′PB (S) motifs shown in *B*. Enzymes are EST, estrogen 17-β sulfotransferase (*Mus musculus*; Protein Data Bank [PDB] code: 1AQU); HS3OST-1, heparan sulfate (3-O) sulfotransferase (*M. musculus*; PDB code: 1S6T); HS2OST, heparan sulfate (2-O) sulfotransferase (*Homo sapiens*; PDB code: 3F5F); HS6OST-3, heparan sulfate (6-O) sulfotransferase (*Danio rerio*; PDB code: 5T03); animal TPST (*H. sapiens*; PDB code: 5WRI); bacterial TPST (*Xanthomonas* spp.; GenBank accession number: WP_027703307); and plant TPST (*Arabidopsis thaliana*; BAI22702).

Sulfotransferases share similar globular folds that bind the sulfodonor 3′-phosphoadenosine 5′-phosphosulfate (PAPS) (Fig. 2*A*), the activated intermediate for sulfur assimilation. PAPS synthesis and functions are described elsewhere (52, 53). Two short sequence motifs, termed 5′-PSB and 3′-PB, coordinate the 5′-phosphosulfate and 3′-phosphate groups, respectively (54) (Fig. 2*B*). Sulfotransfer and kinase reactions are similar (Fig. 2*A*), and the geometry of PAPS binding in sulfotransferases is similar to that of ATP binding in nucleotide kinases (55). TPST enzymology has been reviewed elsewhere (56).

Cytoplasmic sulfotransferases termed SULT modify a variety of small molecules such as xenobiotics and hormones, generally share similar overall sequence (57, 58). By contrast, tyrosyl-protein and polysaccharide sulfotransferases share little sequence similarity with one another and have different spacing between the 5′-PSB motif, catalytic base residue, and the 3′-PB motif (Fig. 2, *B* and *C*), reflecting their engagement with different polymeric substrates.

Metazoan tyrosyl-protein and polysaccharide sulfotransferases are anchored to the Golgi lumen through an amino-terminal transmembrane segment and thus are type II transmembrane proteins. These sulfotransferases modify proteins and sulfated glycosaminoglycans (GAGs) in spatial and temporal coordination with other modifications such as protein glycosylation (2, 3, 5, 43, 59, 60). Two TPST isoenzymes are encoded by most genera throughout the Metazoa (5). The human TPST isoenzymes are expressed broadly, with one or the other predominating in certain tissues (43, 61). Understanding these expression patterns requires defining the relationship between isoenzyme expression and the availability and relative affinity of substrates.

For example, homozygous *tpst-1* null mice have lower average body weight, which may result in part from reduced levels of the sulfated hormones cholecystokinin (CCK) and gastrin (described later). By contrast, homozygous *tpst-2* null mice display primary hypothyroidism, likely resulting from failure to sulfate the receptor for thyroid-stimulating hormone (described later). Homozygous *tpst-2* null males also are infertile. Finally, homozygous *tpst-1 tpst-2* double-null mice usually die soon after birth because of cardiopulmonary insufficiency (62, 63, 64, 65, 66). Thus, TPST-1 and TPST-2 functions overlap only partially. The challenge remains to identify specific bases for most of these phenotypes.

Metazoan TPST displays broad substrate specificity. Most substrates with at least moderate affinity have an Asp residue just proximal to the sulfoaccepting Tyr residue (61, 67, 68, 69, 70, 71) (Fig. 1). Nonconserved flanking residues, often rich in acidic Asp and Glu residues, make multiple interactions with different TPST residues near the active site (67, 68, 70). Overall, these broader sequence features and their roles in metazoan TPST-binding affinity and sulfation efficiency remain obscure (72).

Plants encode a single TPST (53). In the model plant *Arabidopsis thaliana*, a null mutant displays developmental phenotypes such as dwarfism (73), consistent with the functions for the only known plant TPST substrates, sulfopeptide hormones involved in multicellular development (described later).

Plant TPST has a carboxyl-terminal transmembrane segment (type I transmembrane protein) in contrast to metazoan TPST and GAG sulfotransferases (73, 74) (Fig. 2*C*). Sequences for plant and metazoan TPST are not obviously related, although plant TPST does share carboxyl-terminal sequence similarity with heparan sulfate 6-O-sulfotransferase (HS6OT), a GAG sulfotransferase (73). This carboxyl terminus is a prominent α-helix in the HS6OT-3 X-ray structure (75) and is not present in other characterized sulfotransferases.

Initial analysis did not detect plant TPST sequence similarity to the conserved 5′-PSB and 3′-PB motifs (73). With more sequences now available, it is evident that different polymer sulfotransferases have PAPS-binding 5′-PSB and 3′-PB motifs with varied sequences and spacings, and so plant TPST sequences do include putative PAPS-binding motifs that match well with those from other polymer sulfotransferases (Fig. 2*B*). Experimental work is necessary to test key residues in the plant TPST putative 5′-PSB and 3′-PB motifs and to examine substrate specificity determinants.

Plant TPST orthologs are conserved throughout green plants including unicellular algae like *Chlamydomonas* spp. that are not known to synthesize sulfopeptide hormones (53, 73). It will be interesting to learn the algal substrate proteins for TPST and to determine if any are conserved in land plants.

**Figure 3:**
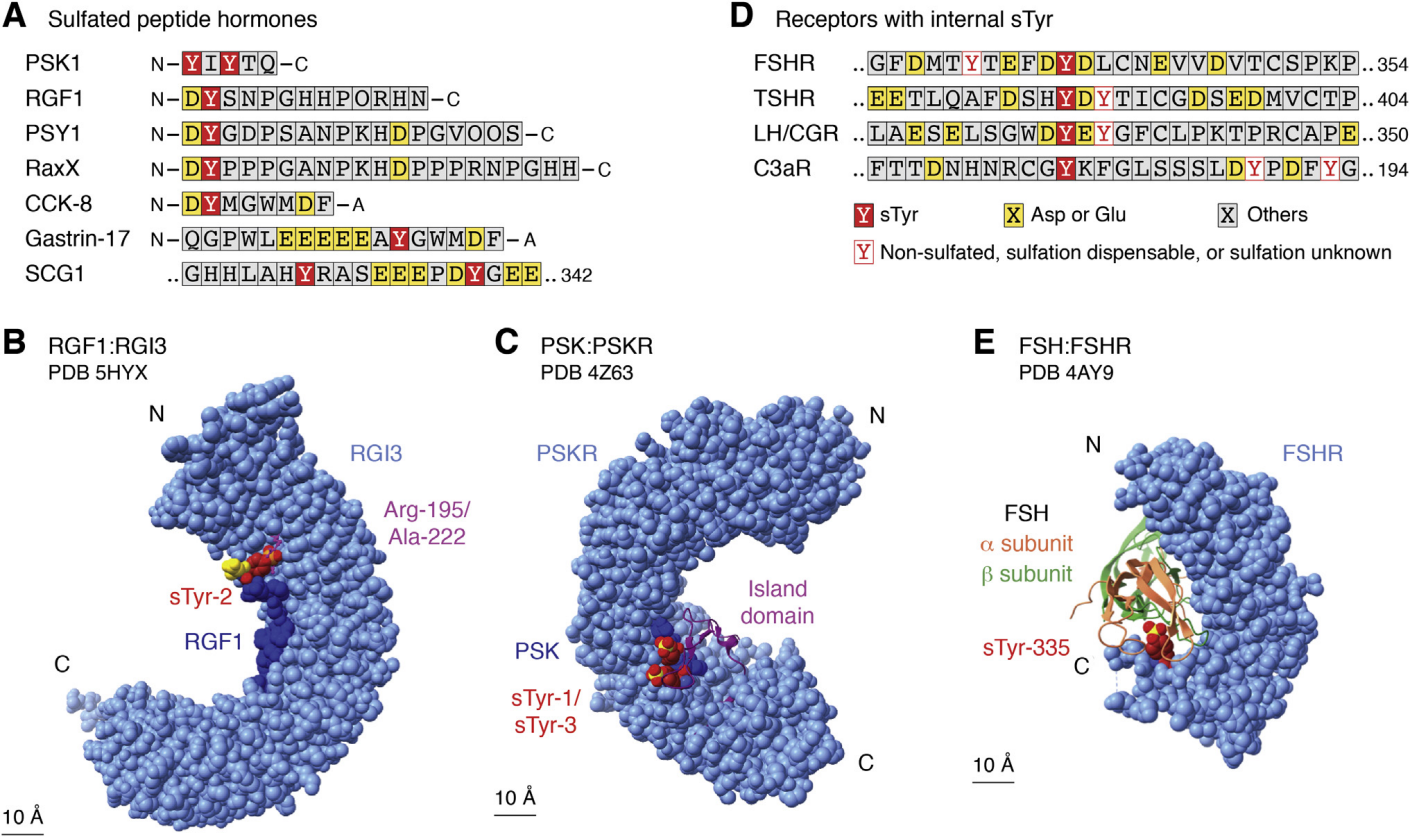
**sTyr in peptides and receptors.***A*, sTyr contexts are shown for the mature forms of the plant sulfopeptide hormones PSK1 (phytosulfokine), RGF1 (RGF), PSY1 (peptide sulfated on tyrosine), the hormone mimic RaxX (required for activating XA21 protein X), and the animal sulfopeptide hormones CCK-8 (cholecystokinin), gastrin-17, and one of two sulfopeptides derived from the secretogranin-1 (SGC-1) precursor. Residues are colored according to the key in *D*. O denotes hydroxyproline. *B*, the cocrystal X-ray model of sulfated RGF (*dark blue*) bound to the RGI3 (RGF-insensitive-3) receptor ectodomain (*light blue*) is depicted at 2.6 Å resolution (86) (Protein Data Bank code: 5HYX). Atoms are shown as van der Waals spheres. The RGI3 ectodomain is built from 23 LRRs (leucine-rich repeats) stacked one upon the next. RGF residue sTyr-2 (*dark red*; S atom, *yellow*) contacts RGI3 residues Arg-195 and Ala-222, depicted as *balls and sticks* (*magenta*). RGF residue Asp-1 (*yellow*) is mostly exposed. *C*, the cocrystal X-ray model of sulfated PSK (*dark blue*) bound to the PSK receptor ectodomain (*light blue*) is depicted at 2.5 Å resolution (88) (Protein Data Bank code: 4Z63). Atoms are shown as van der Waals spheres. The RGI3 ectodomain is built from 21 LRRs stacked one upon the next, with a 36-residue island domain, shown as a *ribbon* (*magenta*), inserted into LRR number 18. PSK residues sTyr-1 and sTyr-3 (*dark red*; S atoms, *yellow*) mostly contact residues in the island domain. *D*, sTyr contexts are shown for internal segments (Fig. 1*A*) of the receptors for FSH (follicle-stimulating hormone), TSH (thyroid-stimulating hormone), LH/CGR (luteinizing hormone/choriogonadotropin), and complement component C3a. *E*, the cocrystal X-ray model of FSH (α subunit, *salmon*; β subunit, *light green*) bound to the sulfated FSH receptor (FSHR) ectodomain (*light blue*) is depicted at 2.5 Å resolution (93) (Protein Data Bank code: 4AY9). The FSHR ectodomain is built from 12 LRRs stacked one upon the next. FSHR residue sTyr-335 (*dark red*; S atom, *yellow*) contacts several residues each in both FSH subunits. sTyr, sulfotyrosyl residue.

Most bacteria, archaea, and nonphotosynthetic eukaryotic microbes do not encode TPST. Nevertheless, TPST is made by some species of plant-pathogenic bacteria in the genus *Xanthomonas* that also synthesize the RaxX protein (required for activation of Xa21-mediated immunity), a molecular mimic of the PSY (plant peptide containing sulfated tyrosine) sulfopeptide hormone (76, 77) (Fig. 3*A*). The bacterial TPST sequence is most similar to that of the Golgi-localized metazoan TPST, except that bacterial TPST acts in the cytoplasm prior to substrate secretion and therefore is not membrane anchored (78).

## sTyr increases binding affinity for peptide hormone–receptor pairs

Secreted sulfopeptide hormones of roughly 10 to 20 residues control growth and development in land plants (38, 79, 80, 81), as well as digestion, neurotransmission, and endocrine functions in animals (82, 83). Most plant sulfopeptide hormones possess the invariant amino-terminal residue pair Asp-1 sTyr-2, where the Asp residue presumably directs plant TPST to the substrate Tyr residue (Figs. 1*A* and 3*A*).

Plant peptide hormones bind their cognate receptors through leucine-rich repeat (LRR) extracellular domains (84, 85). The plant sulfopeptide hormones tested display high affinities for their cognate receptor LRR domains, ranging from 1 to about 300 nM, and in each case, the sTyr contributes 10-fold or more to affinity compared with the nonsulfated peptide (48, 78, 86, 87).

Plant receptor binding to the sTyr is shown in X-ray cocrystal structures for the sulfopeptide hormones root meristem growth factor (RGF) and Casparian strip integrity factor with their cognate receptor LRR domains (86, 87). Both structures reveal overall hydrophobic sTyr-binding interfaces, but only one Arg residue is conserved among residues that make specific contacts (Fig. 3*B*). Based on these two examples, it appears that plant LRR receptor sTyr-binding interfaces have few sequence constraints and therefore might readily be formed during evolution. Further examples of LRR–sulfopeptide interactions are needed to determine the limits of these constraints.

The pentapeptide phytosulfokine (PSK) is the only known plant hormone that contains two sTyrs (Fig. 1*A*) which together confer about 40-fold higher affinity for the PSK receptor (38, 88, 89). The PSK receptor LRR domain contains a 36-residue “island” that contacts both sTyrs through multiple interactions (88) (Fig. 3*C*). These different features illustrate how sTyrs are versatile interaction determinants even in a broadly similar context such as sulfopeptide–LRR binding.

The animal sulfopeptide hormones CCK and gastrin are processed to generate different length bioactive peptides with a common carboxyl-terminal Phe-amide (Figs. 1*A* and 3*A*). The CCK sTyr mostly is sulfated (83), whereas the gastrin sTyr is heterogeneously sulfated, perhaps because of a low-affinity sequence environment for TPST recognition (67).

CCK and gastrin signal through the homologous G protein–coupled receptors (GPCRs) CCK1R and CCK2R. CCK1R binds sulfated CCK with up to 1000-fold greater affinity than nonsulfated CCK, sulfated gastrin, or nonsulfated gastrin. In contrast, the CCK2R makes little distinction (90). Thus, ligand sulfation state is a specificity determinant for CCK1R–CCK interaction. No X-ray structure is available for either CCK1R or CCK2R, but various studies indicate that these sulfopeptides interact across a broad region of the receptor external face (82), and that a conserved Arg residue in a CCK1R extracellular loop interacts specifically with the CCK sTyr (91).

Mirroring these interactions between nonsulfated receptors and sulfated peptide hormones, some nonsulfated ligands bind single sTyr-containing receptors (Fig. 3*D*). Glycoprotein hormones such as follicle-stimulating hormone are central to the complex endocrine system that regulates normal growth, sexual development, and reproductive function. Receptors for glycoprotein hormones comprise an amino-terminal LRR domain connected through a flexible region to a carboxyl-terminal G protein–coupled receptor (Figs. 1*A* and 2*A*). This interdomain region contains an sTyr that is indispensable for hormone recognition and signaling (92, 93).

Upon binding to the LRR domain, follicle-stimulating hormone (about 200 residues) exposes a hydrophobic interface that contains two positively charged residues for electrostatic interactions with the sTyr sulfate (92, 93) (Fig. 3*E*). Homologous receptors for thyroid-stimulating hormone and leutenizing hormone similarly contain an essential interdomain-flexible region with a single sTyr (94) (Fig. 3*D*).

Component C3a, a 77-residue peptide generated through the complement cascade, acts through its receptor to stimulate many aspects of the inflammatory response, sometimes leading to anaphylaxis (95). The GPCR C3a receptor has an sTyr, essential for binding C3a, within an unusually large extracellular loop (96) (Figs. 1*A* and 3*D*). To date, an atomic-level view of receptor binding and activation is not available (97).

## sTyr clusters mediate a variety of protein–protein interactions

In most cases, sTyrs occur in clusters of two or three within a six-residue span. This arrangement enables a wide range of interactions as illustrated by these examples.

### sTyr clusters are essential for HIV-1 entry

HIV-1 binding and entry depends on the viral envelope glycoprotein (gp) spike, a trimer of glycoprotein spike 120 kDa subunit (gp120)–gp41 heterodimers (98). First, the spike binds to the cell-surface receptor CD4, thereby altering gp120 conformation to expose its bridging sheet element (99). Then, the coreceptor amino terminus binds the bridging sheet, triggering membrane fusion and viral entry (98, 100, 101).

**Figure 4:**
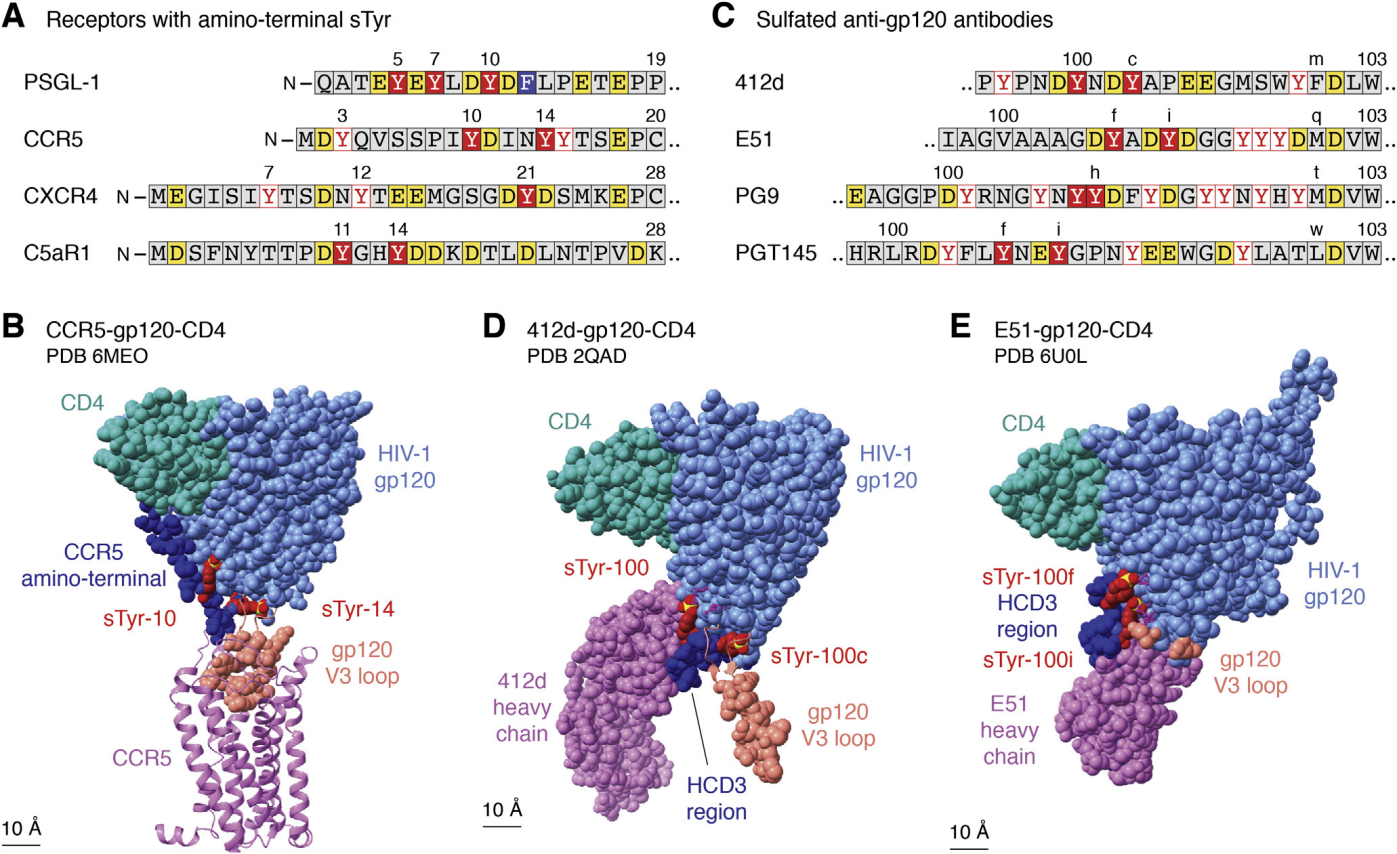
**sTyr in receptor aminotermini and in anti-gp120 antibodies.***A*, sTyr cluster contexts are shown for the aminotermini of the cell-surface proteins PSGL-1 (P-selectin glycoprotein ligand-1), CCR5 (CC-type chemokine receptor 5), CXCR4 (CXC-type chemokine receptor 4), and C5aR (complement component C5a receptor). Residues are colored according to the key for Figure 3*D*. *B*, the cryo-EM model of HIV-1 gp120 (glycoprotein 120; *light blue*) bound to host CCR5 (*light purple*) and one subdomain of host CD4 (CD4 receptor; *light green*) is depicted at 3.9 Å resolution (101) (Protein Data Bank [PDB] code: 6MEO). Atoms are shown as van der Waals spheres except for CCR5, depicted as *ribbons* the better to display the seven transmembrane helix structure and to visualize interaction with the gp120 V3 loop residues (*salmon*). The CCR5 amino-terminal segment (*dark blue*) includes residues sTyr-10 and sTyr-14 (*dark red*; S atoms, *yellow*) that interact with residues in the gp120 bridging sheet element including the base of the V3 loop. Certain residues at the base of the V3 loop are shown as *ribbons* the better to visualize the sTyrs. *C*, sTyr cluster contexts are shown for the HCD3 regions from representative CD4i anti-gp120 antibodies: 412d (PDB code: 2QAD); E51 (PDB code: 6U0L); PG9 (PDB code: 3U2S); PGT145 (PDB code: 3U1S). *D*, the cocrystal X-ray model of HIV-1 gp120 (glycoprotein 120; *light blue*) bound to the antibody 412d heavy chain (*light purple*) and one subdomain of host CD4 (*light green*) is depicted at 3.3 Å resolution (112) (PDB code: 2QAD). Certain residues at the base of the V3 loop are shown as *ribbons* the better to visualize the sTyrs. The HCD3 region (*dark blue*) includes residues sTyr-100 and sTyr-100c (*dark red*; S atoms, *yellow*) that interact with residues in the gp120 bridging sheet element including the base of the V3 loop. Certain residues at the base of the V3 loop are shown as *ribbons* the better to visualize the sTyrs. Only a portion of the 412d light chain is shown. *E*, the cryo-EM model of HIV-1 gp120 (*light blue*) bound to the antibody E51 heavy chain (*light purple*) and one subdomain of host CD4 (*light green*) is depicted at 3.3 Å resolution (117) (PDB code: 6U0L). The HCD3 region (*dark blue*) includes residues sTyr-100f and sTyr-100i (*dark red*; S atoms, *yellow*) that interact with residues in the gp120 bridging sheet element. The V3 loop is not resolved in this structure; gp120 residues at the V3 base (*salmon*) indicate its position. Only a portion of the E51 light chain is shown. sTyr, sulfotyrosyl residue.

The principal T-cell coreceptors for HIV-1 entry are the chemokine receptors CCR5 and CXC-type chemokine receptor 4 (CXCR4) (102). These are GPCRs for chemokines, secreted signaling proteins of approximately 75 residues that direct leukocyte movement toward injury or infection sites (103). The amino-terminal segment in many chemokine receptors includes an sTyr cluster that is essential for high-affinity chemokine binding (44, 46, 104, 105, 106, 107) (Figs. 1*B* and 4A). Interest in sTyr function accelerated with discoveries that the CCR5 sTyr cluster is essential for HIV-1 entry (100) and is mimicked in some HIV-1 broadly neutralizing antibodies (108).

CCR5 residues sTyr-10 and sTyr-14 are necessary for proper gp120–CCR5 interaction, whereas sulfation at residues Tyr-3 or Tyr-15 was dispensable (109, 110, 111) (Fig. 4*A*). A cryo-EM structural model shows the CCR5 amino-terminal segment in an extended conformation across the gp120 bridging sheet. In this model, residue sTyr-14 is mostly buried in a pocket lined with hydrophobic contacts and capped by electrostatic interactions. By contrast, residue sTyr-10 is mostly exposed (98, 101) (Fig. 4*B*).

Certain HIV-1-infected individuals produce antibodies that recognize the CD4-induced (CD4i) conformation of gp120 with the bridging sheet exposed (108, 112). Most of these CD4i antibodies contain heavy chains with unusually long and diverse sequences for the third complementarity-determining regions (HCD3) that form protruding loops to contact otherwise poorly accessible epitopes (112, 113, 114). Many of these CD4i antibody HCD3 sequences include sTyrs required for gp120 binding (108, 112) (Fig. 4*C*).

In an X-ray cocrystal structure with the gp120–CD4 complex, residues sTyr-100c^412d^ and sTyr-100^412d^ bind the same gp120 interfaces as the modeled residues sTyr-10^CCR5^ and sTyr-14^CCR5^, respectively (101, 115) (Fig. 4*D*). (The antibody numbering accounts for variable numbers of residues, labeled 100a–100w, between HCD3 residues at positions 100 and 101.) Separate studies with CCR5 sulfopeptides and antibody 412d sulfoforms expressed with the expanded genetic code reveal that the buried residues sTyr-14^CCR5^ and sTyr-100c^412d^ are essential for binding, whereas the more exposed residues sTyr-10^CCR5^ and sTyr-100^412d^ are ancillary (111, 116). Thus, the CCR5 and 412d sTyr-containing elements apparently share similar interaction with gp120.

A cryo-EM structural model shows a different binding pattern by antibody E51 (117) (Figs 1*B* and 4*E*). Although residue sTyr-100i^E51^ binds to the same gp120 interface as the exposed ancillary residues sTyr-10^CCR5^ and 412d sTyr-100^412d^, residue sTyr-100f^E51^ instead makes unique interactions (Fig. 4*E*). Interestingly, for antibody E51 sulfoforms, exposed residue sTyr-100i^E51^ is essential, whereas the unique residue sTyr-100f^E51^ is ancillary (32).

Thus, antibody E51 sTyr–gp120 interactions are distinct from those for chemokine receptor CCR5 and antibody 412d. Intriguingly, E51 also is more potent than 412d (117) and forms the basis for an effective anti-HIV peptide (118). Apparently, the gp120 bridging sheet is versatile enough to make high-affinity interactions with different sTyr-containing flexible segments, increasing opportunities to meet the stereochemical constraints to binding sulfate (112).

### sTyr clusters contribute to combinatorial receptor–peptide interactions

Human cells express about 50 chemokines and about 20 chemokine receptors acting in different combinations in different tissues (119), so binding interface versatility is a hallmark. Roughly, half of the chemokine receptors contain sTyrs in their aminotermini (44). The sTyr cluster forms the core for binding the chemokine ligand globular domain and helps enable a given receptor to interact with multiple chemokines (46, 104, 107, 119). The CCR5 amino-terminal segment has yet to be captured in a receptor-chemokine X-ray or cryo-EM structure (44, 101, 107, 120, 121), suggesting that it is intrinsically disordered (122).

Indeed, NMR spectroscopy and modeling suggests that CCR5–CC-type chemokine ligand 5 (CCL5) interactions are highly dynamic (121, 123). In accordance with these observations, separate sulfopeptide-binding experiments revealed that any combination of two sTyrs at CCR5 positions 10, 14, and 15 enables strong binding to chemokine CCL5 (124). Together, these results support the hypothesis that the CCR5-flexible sTyr-containing anionic segment makes a variety of dynamic yet high-affinity contacts to the chemokine, in striking contrast to the relatively fixed contacts observed in the CCR5–gp120 interactions described previously (121, 124).

Chemokines also bind sulfated GAGs to form chemotactic gradients (103). Notably, GAG sulfate groups make electrostatic interactions with many of the same chemokine basic residues that contact sTyr sulfates. Thus, chemokine functionality is expanded by a single versatile interface that binds different sulfated polymers for different purposes (106, 123, 125).

Chemokines provide signals for leukocyte movement toward sites of infection or injury. This rolling cell adhesion is mediated in part by P-selectin glycoprotein ligand-1 (PSGL-1), a homodimeric mucin-like cell surface glycoprotein. PSGL-1 contains three sTyrs in its amino-terminal flexible segment (Figs. 1*B* and 4*A*) and engages with the cell-surface adhesion P-selectin through a mechanosensitive catch bond that enables rapid engagement and release under force in the bloodstream flow (126, 127). An X-ray cocrystal structure of a short PSGL-1 sulfoglycopeptide bound to the P-selectin amino-terminal domain shows residue sTyr-7 with several electrostatic and hydrophobic contacts to residues in P-selectin and residue sTyr-10 with multiple hydrophobic contacts that orient the sulfate for hydrogen bonding to a critical Arg residue (2, 128).

Although all three PSGL-1 sTyrs are necessary for full P-selectin interactions, any one sTyr suffices for partial function in a variety of assays relevant to rolling cell adhesion (129). Indeed, residue sTyr-5 is not visible in the P-selectin–PSGL-1 cocrystal structure, suggesting that the observed structure may represent one of several productive conformations (128). In this hypothesis, the sTyr cluster potentially presents a variety of conformations suitable for interaction. Nevertheless, a separate study identified sTyr-7 as essential for PSGL-1 binding to P-selectin (130), congruent with the extensive interactions made by this residue (128).

Separately, PSGL-1 helps regulate aspects of T-cell function, including the progressive loss of effector function in so-called exhausted T cells that accompany persistent antigen stimulation (131). In this context, PSGL-1 is the receptor for V-domain immunoglobulin suppressor of T-cell activation (VISTA), a pH-responsive T-cell inhibitor (131). In a computational model, all three PGSL-1 sTyrs make ionic interactions with His residues along one edge of VISTA. In this model, sTyr interaction depends upon His protonation, as would occur in acidic tumor microenvironments (pH ≤ 6) (49). Sulfation is critical for VISTA–PSGL-1 binding, but contributions of individual sTyrs are not known (49). Together, interactions with P-selectin and VISTA illustrate how sTyr cluster structural flexibility enables impressive functional versatility by the PSGL-1 amino-terminal segment.

Complement protein C5a (74 residues), generated through the complement cascade, stimulates inflammatory mediator release and also is a potent chemoattractant (95). Similar to chemokine receptors, the G protein–coupled C5a receptor amino-terminal segment contains two sTyrs (132) (Figs. 1*B* and 4*A*). Chemotaxis inhibitory protein of *Staphylococcus aureus* (CHIPS; 121 residues) also binds the amino-terminal segment, thereby inhibiting C5a-dependent inflammatory responses (97). The C5a receptor sTyr pair increases CHIPS binding affinity for peptides from the C5a receptor amino-terminal segment by approximately 1000-fold (133). In the NMR structure of CHIPS bound to the C5a receptor amino-terminal peptide, the two sTyrs are within a five-residue β-strand and make several hydrophobic and electrostatic contacts to residues in CHIPS (133).

These three examples—CCR5, PSGL-1, and C5aR—illustrate a variety of sTyr interactions ranging from relatively ordered, as with the sulfoantibody–gp120 and C5aR–CHIPS interactions, to highly dynamic, as with the CCR5–CCL5 and PSGL-1–P-selectin interactions. Thus, it appears that two sTyrs can enable a wider range of binding modes and partners than observed in the single sTyr hormones and receptors described previously.

## sTyr clusters in hirudin-like motifs bind the blood clotting enzyme thrombin

The sTyr cluster–containing anionic segments described previously are versatile and dynamic, and there is no obviously conserved sequence pattern. Here, a different collection of anionic segments makes conserved interactions and contain conserved sTyr cluster sequences. Thus, sTyr clusters participate across a range of anionic segment functions.

Aberrant hemostasis—ranging from profuse bleeding to excessive clotting—underlies or complicates several human conditions, and many treatments have been developed (134, 135, 136). The serine endoprotease α-thrombin is central to controlling the balance between initiating and terminating blood clotting (thrombosis). Thrombin function depends on its interactions with molecular tethers in several different substrates and inhibitors (137, 138) (Fig. 1*C*). This number and diversity of thrombin-binding partners provides an opportunity to explore unity and diversity in sTyr cluster interactions.

**Figure 5:**
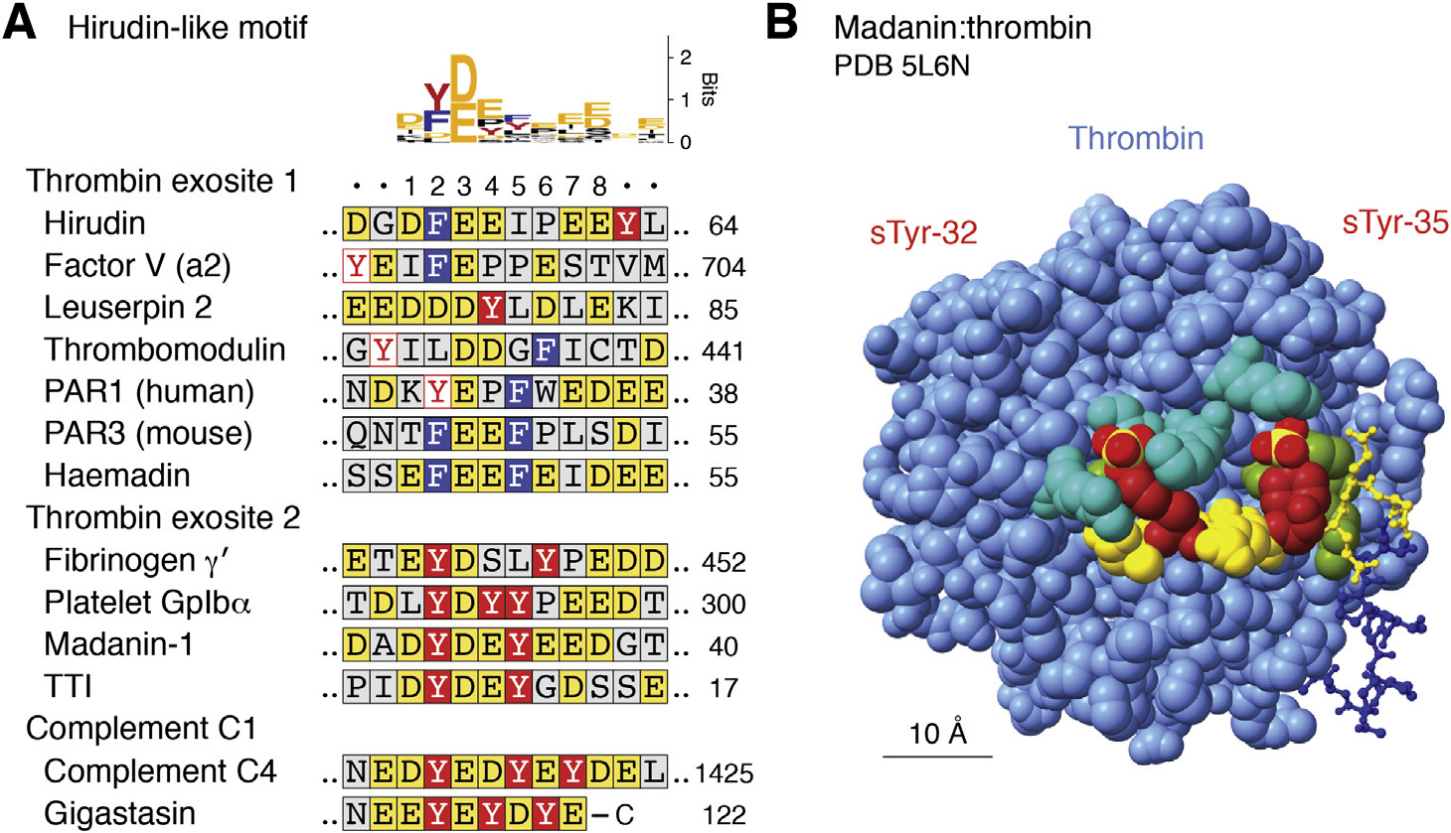
**sTyr in hirudin-like motifs.***A*, sTyr cluster contexts are shown for thrombin-binding hirudin-like motifs from hirudin (Protein Data Bank [PDB] code: 2PW8); factor V (a2 segment; PDB code: 3P6Z); leuserpin 2 (PDB code: 1JMO); thrombomodulin (PDB code: 1DX5); peptide-activated receptor-1 (PAR1; human; PDB code: 3LU9); peptide-activated receptor-3 (PAR3; mouse; PDB code: 2PUX); haemadin (PDB code: 1E0F); madanin-1 (PDB code: 5L6N); fibrinogen γ' (PDB code: 2HWL); platelet GpIbα (PDB code: 4CH2); tsetse thrombin inhibitor (TTI; PDB code: 6TKG); complement C4 (GenBank accession number: AAA51855); and gigastasin (PDB code: 5UBM). Residues are colored according to the key for Figure 3*D*. The sequence logo (180, 181) was generated from the alignment shown. The hirudin-like motif is defined here as positions 1 to 8. Positions 2 and 3 comprise a conserved aromatic-acidic residue pair, consisting of Phe, Tyr, or sTyr at position 2 and Asp or Glu at position 3. The remaining hirudin-like motif residues are hydrophobic, negatively charged, or both in the case of sTyr, and binding to thrombin exosites involves both electrostatic and hydrophobic contacts (142). *B*, the cocrystal X-ray model of sulfated madanin bound to exosite 2 on thrombin (*light blue*) is depicted at 1.6 Å resolution (144) (PDB code: 5L6N). Atoms are shown as van der Waals spheres, except for madanin residues 36 to 60 shown as *balls and sticks*. Madanin residues sTyr-32 and sTyr-35 (*dark red*; S atoms, *yellow*) make electrostatic interactions with exosite 2 Arg and Lys residues (*light green*) and hydrophobic interactions with other residues (*olive green*). Madanin Asp and Glu residues (*yellow*) are highlighted for reference. sTyr, sulfotyrosyl residue.

One clot-inhibiting therapeutic used for over 100 years is hirudin, an sTyr-containing direct thrombin inhibitor (DTI) made by *Hirudo* leeches (139, 140). Hirudin (65 residues) comprises a carboxyl-terminal anionic segment that binds thrombin and an amino-terminal domain that occludes the thrombin active site (Fig. 1*C*). Similar anionic segments, termed hirudin-like motifs (141), are present in other thrombin-binding proteins (137, 138) (Fig. 5*A*).

Thrombin contains two cationic surface patches, termed anion-binding exosites, that bind different protein substrates through their hirudin-like motifs (137, 138). Thrombin exosite 1 aligns both endoprotease substrates and DTIs such as hirudin with respect to the adjacent active site, whereas exosite 2 tethers thrombin at the site of blood clots, binding platelet surfaces *via* platelet glycoprotein Ibα and to fibrin clots *via* fibrinogen γ' (142, 143) (Fig. 1*C*). Exosite 2 also binds the DTIs madanin and tsetse thrombin inhibitor (51, 144). Each exosite contains several basic Arg and Lys residues, enabling different contacts with acidic Asp, Glu, and sTyrs in different hirudin-like motif sequences.

As defined by the sequence alignments presented in Figure 5*A*, the hirudin-like motif spans eight contiguous residues, of which most are acidic, aromatic, or both in the case of sTyr. Critically, hirudin-like motifs in all four proteins known to bind thrombin exosite 2 contain an sTyr cluster, whereas hirudin-like motifs from exosite 1-binding proteins, including hirudin itself, rarely contain sTyrs and none at conserved positions (Figs. 1*C* and 5*A*).

Cocrystal X-ray structures of thrombin with exosite 2-bound sulfopeptides, illustrated here with the DTI madanin (144), reveal conserved contacts to thrombin residues across exosite 2 (24, 51, 145) (Fig. 5*B*). Madanin residues sTyr-32 and Asp-33, which occupy the hirudin-like motif conserved positions 2 and 3, make both ionic and nonpolar contacts to numerous exosite 2 residues (144) that form the core interaction with thrombin (24). Madanin residue sTyr-35 is more exposed (Fig. 5*B*). Thrombin contacts to all three residues are conserved in all four available cocrystal X-ray structures (51), indicating that the sTyr cluster–containing hirudin-like motif provides a well-defined high-affinity binding determinant for exosite 2.

Peptide-binding analyses with sulfopeptides (51, 144) or phosphopeptides (24, 145) illuminate these structural models, again illustrated here with sulfomadanin. The thrombin inhibition constant for doubly sulfated madanin is about 400-fold lower than for nonsulfated madanin, showing the essentiality of sTyr. Madanin with only sTyr-35 is twofold more effective than nonsulfated madanin, whereas madanin with only sTyr-32 is about 25-fold more effective (144). Thus, like chemokine receptor CCR5 interaction with gp120, the core sTyr (sTyr-32^madanin^; sTyr-14^CCR5^) is essential, whereas the ancillary sTyr (sTyr-35^madanin^; sTyr-10^CCR5^) enhances binding only in the presence of the core (111, 144) (Fig. 5*B*).

Like hemostasis, the complement system is activated through an endoprotease cascade (146). Complement component C1, a serine protease homologous to thrombin, has an anion-binding exosite that interacts with an sTyr cluster–containing hirudin-like motif in the substrate C4 (147, 148) (Fig. 5*A*). The X-ray cocrystal structure of complement C1 bound to gigastasin, a C1 inhibitor made by *Haementaria* leeches, reveals electrostatic contacts from C1 exosite basic residues to sulfate O atoms from gigastasin residues sTyr-117 and sTyr-119 (149). Thus, the homologous hemostasis and complement systems share the use of hirudin-like motifs to direct partner protein binding to exosites.

Segments with sTyr clusters are prevalent in bloodstream activities, including leukocyte migration and signaling, blood clotting, complement activation, and triglyceride metabolism. These activities also are modulated extensively by interactions with sulfated GAGs such as heparan, anionic oligosaccharides that help control hemostasis through binding thrombin (103, 150, 151, 152). Indeed, thrombin exosite 2 was identified initially as the binding site for the highly sulfated GAG heparin (142). Thus, a cluster of two or three sTyrs can resemble a sulfated GAG to make conserved contacts with some of the many available basic residues in exosite 2, thereby expanding its valency beyond that of heparin binding. Notably, basic exosite residues that contact GAG substrates (142) mostly are distinct from those that contact sTyr-containing substrates, illustrating the broad binding versatility provided by exosite 2.

## sTyr in acidic arrays

Finally, some sTyrs lie within relatively long tracts of acidic Asp and Glu residues (Fig. 1*D*). Glycosylphosphatidylinositol-anchored high-density lipoprotein binding protein 1 (GPIHBP1) is a membrane protein critical for triglyceride metabolism. The GPIHBP1 amino-terminal acidic tract requires a single sTyr for proper interaction and function with its binding partner, lipoprotein lipase (47). As with other aforementioned examples, the GPIHBP1 amino-terminal acidic tract is not visible in the lipoprotein lipase–GPIHBP1 cocrystal X-ray structure (153).

At the opposite extreme lie certain extracellular matrix proteins such as fibromodulin, whose amino terminus has an extended tract of acidic residues including several sTyrs (154, 155) (Fig. 1*D*). This region binds collagen and a variety of heparin-binding proteins (156, 157). The collagen-modifying enzyme lysyl oxidase has a similar sTyr-containing acidic tract through which it binds collagen (158). The fibrinogen and lysyl oxidase acidic tracts contain several Tyr residues, but in most cases, it has not been possible to determine which of these is sulfated to form sTyr (157, 158).

## Challenges and prospects

The examples presented here exemplify the diverse contexts for the sTyr PTM. A single sTyr can enhance binding affinity by several 100-fold. A cluster of two or three sTyrs provides a wider range of binding modes, from versatile, as illustrated by the chemokine receptor CCR5 sTyr-containing amino-terminal segment, to stringent, as illustrated by hirudin-like motif interactions with thrombin exosite 2.

PTMs often work in concert, with different combinations exerting different effects (1). sTyrs often occur with nearby *N*- or *O*-linked glycans, and differential glycosylation can affect function (2). For example, PSGL-1 residue Thr-16, near the sTyr cluster (Fig. 4*A*), is glycosylated in leukocytes to enable interaction with P-selectin, but it is not glycosylated in certain T cells wherein PSGL-1 interacts with VISTA (49, 159). This encourages a parallel hypothesis, that differential tyrosyl sulfation also can help determine binding partner selection. Indeed, the chemokine receptor CXCR4 (for one example) requires residue sTyr-21 for chemokine ligand CXCL12 binding but not for HIV-1 entry (160). It is not known if or how CXCR4 tyrosine sulfation is regulated with respect to cell type or external stimulus.

The extent of sulfation at a given Tyr residue can be incomplete, such that only a fraction of proteins in a given population carry a particular sTyr (19, 44). This is difficult to evaluate directly because MS analyses may result in sulfate loss (9). Does heterogeneous sulfation result from stochastic TPST catalysis? Or are different sulfation states (potentially with distinct ligand-binding properties) programmed in different cell types or in response to different stimuli?

Evaluating the location and extent of tyrosine sulfation also requires better understanding of how TPST engages its substrates and how its activity is regulated. Recently described TPST inhibitors may prove helpful (161). Substrates require nearby Asp or Glu residues for efficient sulfotransfer, but overall, specificity determinants are not well defined (70, 71, 72) in comparison to tyrosine kinases (162). However, animal cells make hundreds of tyrosine kinases but only two TPSTs. Therefore, TPST might catalyze some level of indiscriminate sulfation within a flexible anionic segment containing multiple Tyr residues. For example, several nearby Tyr residues within glycopeptide receptors and the C3a receptor are sulfated *in vivo* (Fig. 3*D*), even though sulfates at these positions are not required for receptor function (94, 96).

Similarly, chemokine receptor CCR5 residue sTyr-3 (Fig. 4*A*) is considered to have minimal functional significance (109, 110, 163, 164) and therefore usually is not studied (111, 124). However, *in vitro* studies show that position Tyr-3 is the first to be sulfated in a CCR5 peptide that includes residue Met-1 (165, 166). Does sTyr-3 result simply from “bystander” sulfation by TPST enzymes with broad substrate recognition? Or does it have a defined function (19)?

*In vitro*, pTyr can substitute for sTyr in some contexts (24, 39, 40) but not in others (164). Nevertheless, stringent specificity for sTyr *in vivo* is seen from the number of molecular mimics that contain sTyr, including anti-gp120 CD4i antibodies (115, 117) as well as inhibitors of chemokine signaling (50), thrombin (26, 51, 144, 167), and complement C1s (149). A striking example is the bacterial RaxX sulfopeptide, which acts as a molecular mimic of the plant PSY sulfopeptide hormone (76, 77) (Fig. 3*A*). Although genes encoding TPST are ubiquitous in Metazoa and plants, they are only sparsely distributed among relatively few bacterial lineages (168) and often are adjacent to genes encoding synthesis and export of extracytoplasmic proteins (V. Stewart and P.C. Ronald, unpublished observations). Thus, because RaxX requires sTyr for activity, the bacterium must synthesize TPST.

One potential research goal is to make defined sTyrs to facilitate engineering of new interaction determinants. An initial proof of principle is eCD4-Ig, a chimeric protein that effectively blocks HIV-1 entry in rhesus macaques (118, 169, 170). eCD4-Ig contains an essential sTyr-rich peptide, derived from the sTyr-containing anti-gp120 antibody E51 variable region (Fig. 4*C*) and therefore is modeled closely after a pre-existing sTyr-containing segment.

More challenging will be to design sTyr interactions *de novo*. Placing sTyrs at predetermined locations would require understanding and manipulating the substrate-binding site as described previously, or it could be accomplished through genetic code modification (29, 32, 33). Designing an effective sTyr-binding site might be more feasible, given the wide range of naturally occurring sites characterized to date. An alternative approach is to use SH2 domains modified to recognize sTyr in place of pTyr (21).

A separate platform for engineering sTyrs may come from ribosomally synthesized and post-translationally modified peptides (RiPPs), microbial natural products with diverse chemistry and potential applications (171). RiPPs are matured through a variety of PTMs, and there is interest in engineering these modifications to create new RiPPs. The bacterial RaxST TPST, which synthesizes the sTyr in the RaxX RiPP, is a good candidate for strategies to introduce novel sTyrs in engineered RiPPs (78, 171). In addition, certain polyketide biosynthesis complexes include SULT-type sulfotransferases among several modifying enzymes (172, 173, 174), so it may be possible to add TPST modules to nonribosomal peptide synthesis complexes (175).

Finally, sTyrs touch many topics in human medicine. For example, there are numerous sTyrs in the homologous multidomain proteins, coagulation factors VIII (FVIII; six sTyrs) and V (seven sTyrs) (176). Classic hemophilia results from FVIII deficiency, and one of the many causative *F8* alleles encodes a FVIII missense substitution of Phe in place of sTyr-1680 (176), documenting an important function for sTyr in hemostasis. Indeed, synthetic FVIII used for human therapy contains a full complement of sTyrs (177). Nevertheless, relatively little is known about sTyr function in these critical hemostasis proteins (178, 179). Research in FVIII and factor V surely can benefit from the technical advances that now enable synthesis of sTyrs at defined locations *in vitro* (25, 27) and *in vivo* (29, 32, 33).

sTyrs are versatile interaction determinants, essential not only for many critical interactions but also challenging to study. This means that sTyrs are underdocumented and understudied relative to other PTMs. Nevertheless, the accumulated knowledge base makes it easier to predict and analyze newly discovered sTyrs, and such discovery will be facilitated as MS methods for sTyr detection increasingly are refined. One expects to see accelerated progress, not only in finding new sTyrs but also in understanding functions for currently known examples.

## Conflict of interest

The authors declare that they have no conflicts of interest with the contents of this article.
